# Clinical, epidemiological and virological features of dengue virus infections in vietnamese patients presenting to primary care facilities with acute undifferentiated fever

**DOI:** 10.1016/j.jinf.2010.01.003

**Published:** 2010-03

**Authors:** Khoa T.D. Thai, Hoang Lan Phuong, Tran Thi Thanh Nga, Phan Trong Giao, Le Quoc Hung, Nguyen Van Nam, Tran Quang Binh, Cameron Simmons, Jeremy Farrar, Tran Thinh Hien, H. Rogier van Doorn, Menno D. de Jong, Peter J. de Vries

**Affiliations:** a Division of Infectious Diseases, Tropical Medicine & AIDS, Academic Medical Center, F4-217, P.O. Box 22700, Amsterdam 1100 DE, the Netherlands; b Oxford University Clinical Research Unit, Wellcome Trust Major Overseas Program, Hospital for Tropical Diseases, 190 Ben Ham Tu, District 5, Ho Chi Minh City, Viet Nam; c Center for Infection and Immunity (CINIMA), Academic Medical Center, University of Amsterdam, Amsterdam, the Netherlands; d Division of Medical Microbiology, Academic Medical Center, Amsterdam, the Netherlands; e Department of Virology, Cho Ray Hospital, Ho Chi Minh City, Viet Nam; f Binh Thuan Medical College, Phan Thiet City, Viet Nam; g Tropical Diseases Clinical Research Center, Cho Ray hospital, Ho Chi Minh City, Viet Nam; h Center for Tropical Medicine, Nuffield Department of Clinical Medicine, University of Oxford, Center for Clinical Vaccinology and Tropical Medicine, Oxford, UK

**Keywords:** Dengue, Epidemiology, Prospective study, Polymerase chain reaction, Vietnam

## Abstract

**Objectives:**

To explore clinical and virological characteristics and describe the epidemiology of dengue in patients who presented with acute undifferentiated fever (AUF) at primary health centers (PHC) in Binh Thuan Province, Vietnam.

**Methods:**

A prospective observational study was conducted from 2001 to 2006 to study the aetiology in AUF patients. Demographic and clinical information was obtained, and dengue polymerase chain reaction (RT-PCR) and serology were performed on a random selection of patients.

**Results:**

Three hundred fifty-one serologically confirmed dengue patients including 68 primary and 283 secondary infections were included in this study. In 25% (86/351) dengue virus (DENV) was detected by RT-PCR among which 32 DENV-1, 16 DENV-2, 1 DENV-3 and 37 DENV-4 were identified. The predominant dengue serotype varied by year with seasonal fluctuation: DENV-4 in 2001–2002, DENV-1 and DENV-2 from 2003 to 2006. Primary dengue was more common in children. Higher viraemia levels (*P* = 0.010) were found in primary infections compared to secondary infections. DENV-1 infected patients had higher viraemia levels than DENV-2 (*P* = 0.003) and DENV-4 (*P* < 0.001) infected patients. Clinical symptoms were often seen in adults. Few differences in clinical symptoms were found between primary and secondary infection and no significant differences in clinical symptoms between the serotypes were observed.

**Conclusions:**

Our data provide insight in the epidemiology, clinical profile and virological features of mild symptomatic dengue patients who presented to PHC with AUF in Vietnam.

## Introduction

Mosquito-borne flavivirus infections such as dengue have rapidly spread and are now one of the most important infectious diseases in the world, in terms of morbidity and mortality.^1,2^ It is a public health problem with growing global incidence and geographic distribution to almost all tropical and subtropical regions, and with a transition from epidemic to endemic transmission intensity. Recent estimates indicate that over 3.5 billion people (∼55%) of the world population are living in areas at risk for dengue.^3^

Dengue is caused by an infection with a dengue virus (DENV) and transmitted primarily by *Aedes* spp. mosquito vectors.^4^ Any of the four distinct serotypes (DENV 1–4) can cause dengue fever (DF) or the more severe forms of the diseases: dengue hemorrhagic fever/dengue shock syndrome (DHF/DSS).^5^ The majority of DENV infections are probably asymptomatic, and only a small number of dengue infections (∼5%) will result in severe forms of the disease.^6^ The mechanisms for the variable clinical course are not completely elucidated, but interactions between virus and host immunity and hyperendemicity of multiple serotypes are believed to play an important role in determining the outcome of disease.^7^

In Vietnam, dengue is not only an urban disease but also the high population density and ecological conditions in the rural areas are also favourable for dengue transmission. Binh Thuan, a rural province in southern Vietnam, is highly endemic for dengue.^8^ Dengue usually presents as a nonspecific febrile illness and is rarely recognized as a clinical entity by physicians at primary health centers (PHC).^9^ However, recent studies have suggested that dengue is the most frequent cause of fever in patients who present to the PHC and is responsible for approximately one-third of all patients with fever.^10^ The prevalence of dengue IgG antibodies among primary school children increased from 50% to 90% with increasing age, indicating high, relatively stable, transmission rates over many years. The annual sero-conversion rate among primary school children, corresponding to the annual incidence rate of primary dengue infections, ranged from 12 to 17%.^11,12^

The data presented here are derived from a prospective observational study from March 2001 to March 2006, with enrolment of acute undifferentiated fever (AUF) patients who presented to 12 PHCs and the provincial malaria control center in Binh Thuan province.^9,10^ One of the objectives was to describe the epidemiology and to detect outbreaks of dengue in Binh Thuan province. In dengue endemic regions, outbreaks often do not necessarily reflect an increase in transmission intensity but merely an increased number of patients with complicated dengue, mostly secondary infections after the (re-)introduction of a new serotype.^13^ During the study period no significant outbreaks of dengue were observed, other than the usual seasonal fluctuation. Here, we report PCR results for patients with serologically confirmed dengue and analyze the epidemiology and clinical and virological characteristics with respect to serotype, antibody response and viraemia.

## Material and methods

### Study site and population

The study site was described previously.^9,10^ Binh Thuan Province is located along the south-eastern coast of Vietnam, 150 km northeast of Ho Chi Minh City. It covers 7828 km^2^ and the estimated population was 1,140,429 inhabitants in 2004.

A prospective observational study was conducted from March 2001 to March 2006. In this study, patients with AUF, who presented to the 12 study PHC and at the provincial malaria control station center in Phan Thiet city, were included. Patients were invited to participate after giving informed consent. A standardized questionnaire was taken to collect demographic and clinical information. Serum samples were collected by venous puncture on presentation (acute sample; *t*0) and after 3 weeks (convalescent sample; *t*3). Serum samples were stored at −20 °C at the study sites until monthly transfer to Cho Ray hospital (Ho Chi Minh City, Vietnam), where they were stored at −70 °C.

### Sample selection for serology and PCR

Complete sets of acute and convalescent samples were collected for serology. In 2001 all collected paired sera were tested with dengue ELISA; from 2002 onwards paired samples were randomly selected as two patients per PHC and per month from the total dataset.^10^ Firstly, serum specific anti-dengue IgM- and IgG-ELISA were performed in sera patients with AUF.^14^ Based on the serological results, patients with DENV infection were included in this study. Secondly, RT-PCR was performed in the acute samples of patients with serologically confirmed dengue.

### Dengue diagnostics

Paired serum samples were tested for dengue with direct IgG ELISA and IgM-Capture ELISA (Focus Technologies Inc., Cypress, CA, USA). Details regarding the ELISA and the interpretation of results have been described previously.^14,15^ Briefly, a fourfold increase of antibody concentrations between *t*0 and *t*3 was considered significant. The IgM concentration on *t*3, relative to the IgG concentration on *t*3 was also used as a criterion. Acute primary dengue virus infection was defined as positive IgM on *t*3 with an IgM/IgG ratio on *t*3 greater than one. A positive IgM on *t*3 with an IgM/IgG ratio on *t*3 less than one, or a negative IgM reaction on *t*3 but with a positive IgG *t*3 and a fourfold molar increase of IgG between *t*0 and *t*3 were classified as acute secondary dengue. A negative IgM reaction on *t*3, a positive IgG on *t*3 but without a fourfold increase between *t*0 and *t*3 was classified as “not acute dengue but past infection”, and a case of both negative IgM and IgG on *t*3 was classified as “no dengue”. Dengue NS1 antigen was detected in a subset of serum samples using the *Platelia*™ Dengue NS1 Ag − ELISA (Bio-Rad Laboratories, Inc., Hercules, CA, USA) according to the manufacturer's instructions.^16^ Optical density (OD) was measured at 450/620 nm, using the Evolis™ absorbance reader. Results were expressed as the OD ratio (ODR) between the OD value of the sample and a calibrator sample that is enclosed with every test panel. An ODR ≥ 1.0 was interpreted as positive.

RNA was isolated from serum from the first serum as described elsewhere.^17^ RNA was reversely transcribed, and dengue virus viraemia levels were assessed using an internally controlled, serotype specific, real-time reverse transcriptase polymerase chain reaction (RT-PCR) assay that has been described elsewhere; results were expressed as cDNA equivalents per ml of serum.^18^

### Ethical considerations

The study was approved by the Review Board of Cho Ray Hospital, the provincial health services of Binh Thuan and the Peoples Committees of the participating communities. All patients (or, for children the parents or guardian) gave written informed consent.

### Statistical analysis

All results were summarized in terms of medians and ranges for continuous data and non-parametric tests were used to compare within groups. For dichotomous variables, Fisher's exact test was performed. Viraemia levels were expressed as the median and 25–75% interquartiles (25–75 IR). All calculations were performed using SPSS (version 16.0, SPSS Inc. Illinois). A two-tailed *p*-value of <0.05 was considered as statistically significant.

## Results

### Sample population

A total of 14,595 febrile patients were included. Eighty-three patients did not meet the inclusion criteria and were excluded from further analysis. Paired sera were collected from 8268 febrile patients; 1938 (23.4%) serum pairs were tested with dengue an IgM- and IgG-ELISA. Dengue was serologically confirmed in 382 (19.7%) cases. Of these, RT-PCR was performed in 351 (91.9%) acute samples. DENV was detectable in 86 (24.5%) samples among which 32 were DENV-1, 16 DENV-2, 1 DENV-3 and 37 DENV-4 were detected. Serologic testing by ELISA revealed 68 primary infections and 283 secondary infections. Demographic information on the study population is shown in Table 1.

### Epidemiologic data

#### Occurrence of dengue serotypes

**Figure 1:**
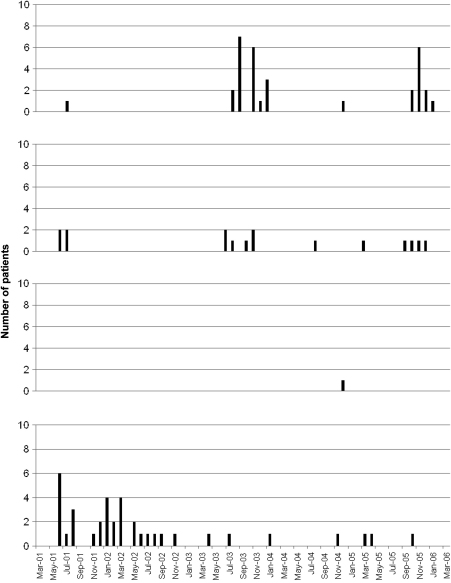
Co-occurrence of multiple dengue virus serotypes in Binh Thuan from 2001 to 2006.

Dengue incidence in Binh Thuan peaks during the rainy season from May to October. DENV-4 was the dominant dengue serotype in 2001–2002. Thereafter, DENV-1 and DENV-2 became the most frequently isolated serotype (Fig. 1).

#### Distribution of primary and secondary infection by age group

**Figure 2:**
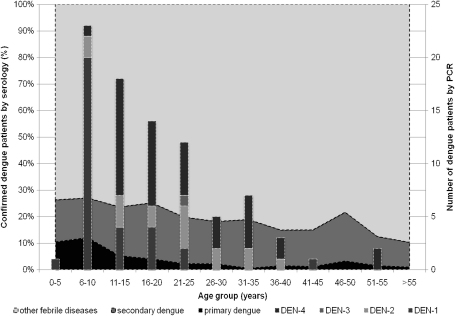
Age group distribution of serologically (*n* = 1938) and RT-PCR (*n* = 86) confirmed dengue virus infection and serotypes among AUF patients in Binh Thuan province, 2001–2006.

When age groups were combined from patients selected for RT-PCR, acute secondary dengue (*n* = 283) was four times as common as acute primary dengue (*n* = 68). The acute primary/secondary dengue ratio in children (<15 years of age) and adults were 0.49 and 0.12 (*P* < 0.001 by Fisher's exact test), respectively. Fig. 2 shows the serotypes which were found in 86 patients and the distribution of serologically confirmed primary and secondary DENV infections stratified by age group.

### Clinical data

#### Differences in clinical presentation between primary and secondary infection

Patients with secondary infection were more likely to be older than those with primary infection (*P* < 0.001). The median time between onset of fever and the first visit was 1 day (25–75 IR, 2 days) for primary and secondary dengue. Myalgia was more frequently reported in secondary infection, whereas gastrointestinal symptoms were more common in primary infections (Table 2).

#### Differences in clinical presentation between primary and secondary dengue with different serotypes

Primary infection was diagnosed in 17 of 32 DENV-1 patients (53.3%) and 1 of 16 DENV-2 patients (6.3%). All DENV-4 patients (*n* = 37) had an antibody response that was compatible with secondary dengue. No significant differences between primary DENV-1 and DENV-2 were observed with respect to the clinical variables. Clinical variables were also not significantly different between secondary DENV-1, DENV-2 and DENV-4 infections.

#### Differences in clinical presentation between children and adults

Table 2 also shows the distribution of clinical variables between children and adults. The median time between onset of fever and the first visit was 1 day (25–75 IR, 1 days) for both children and adults. Symptoms and physical findings were more common in adult patients, such as myalgia, backache, arthralgia, and bruises.

### Virologic data

Viraemia levels were measured in 71 of 86 RT-PCR positive patients (84%); 28 of 32 DENV-1, 11 of 16 DENV-2, 1 of 1 DENV-3 and 32 of 37 DENV-4. The viraemia levels in serum ranged from 1.5 × 10^4^ to 2.7 × 10^11^/mL, with a median of 1.1 × 10^6^/mL (25–75 IR, 4.1 × 10^6^/mL), and were sampled from day 0 to day 4 of fever.

#### Relationship between serum viraemia and antibody response

**Figure 3:**
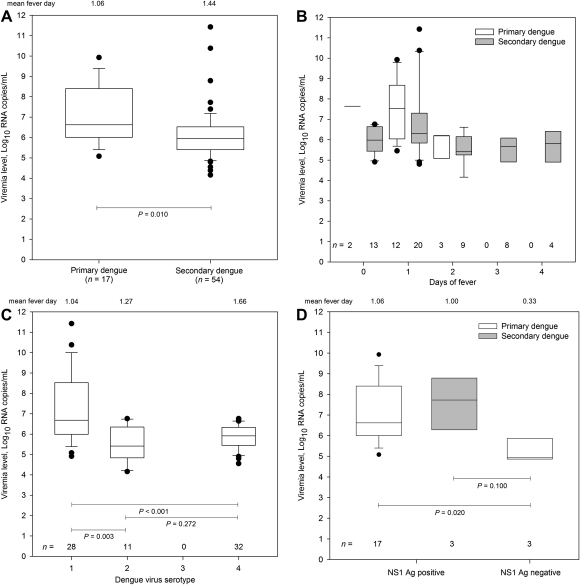
Distribution of viraemia levels in: (A) patients with primary and secondary dengue infections; (B) patients with primary and secondary dengue infection and fever history; (C) patients with DENV-1, DENV-2, DENV-3 and DENV-4 infections; (D) patients with and without NS1 antigen. All comparison within groups was performed using Mann–Whitney tests. Box plots show median values (horizontal line in the box), 25–75% interquartile range (lower-upper limits of the box), 90% range of data (additional bars), and outliers (circles).

Viraemia levels were significantly higher in primary than in secondary dengue (4.2 × 10^6^/mL versus 8.8 × 10^5^/mL, respectively, *P* = 0.010, Fig. 3A). During the first two days of fever, viraemia levels were significantly higher in primary dengue than in secondary dengue (4.2 × 10^6^/mL versus 9.6 × 10^5^/mL, respectively, *P* = 0.036). The distribution of viraemia levels at different time points since illness days in primary and secondary dengue is shown in Fig. 3B.

#### Relationship between serum viraemia and the serotype

DENV-1 infected patients had higher median viraemia levels than DENV-4 infected patients. Viraemia levels in DENV-1 infected patients were higher than in DENV-2-infected patients. Viraemia levels in DENV-2 and DENV-4 infected patients were not significantly different (Fig. 3C). When immune status was taken into account, median viraemia levels remained higher in secondary DENV-1 infected patients than secondary DENV-4 (5.5 × 10^6^/mL versus 8.3 × 10^5^/mL, respectively, *P* = 0.014) and secondary DENV-2 (5.5 × 10^6^/mL versus 2.6 × 10^5^/mL, respectively, *P* = 0.015) infected patients. No association was observed between serum viraemia levels and sex and age.

#### Relationship between serum viraemia and NS1 antigen

NS1 antigen detection was performed in 100 (of 351) acute samples; 40 samples were NS1 antigen positive. DENV was detectable in 31 (78%) of these samples and viraemia levels were measured in 20 samples. DENV was demonstrated in 3 of 60 NS1 antigen negative samples (5.0%). Irrespective of immune status, viraemia levels were higher in NS1 antigen positive patients than NS1 antigen negative patients (1.7 × 10^7^/mL versus 8.6 × 10^4^/mL, respectively) (*P* = 0.016, Mann–Whitney test). Fig. 3D shows differences in viraemia levels among patients with and without NS1 antigen by immune status. The median viraemia levels in patients with secondary DENV infections with NS1 antigen were higher than those without NS1 antigen. Because of the small numbers, this did not reach significance (*P* = 0.100).

## Discussion

In a previous study we showed that dengue is highly endemic in Binh Thuan province in southern Vietnam.^10,19^ Here we show that during a study period of five years, co-circulation of multiple DENV serotypes occurred in this region. Higher viraemia levels were found in primary infections in comparison to secondary infections. DENV-1 infected patients had higher viraemia levels than DENV-2 and DENV-4 infected patients. Clinical manifestations of infections with the different serotypes were similar but symptoms were more commonly observed in adults. Two symptoms differed significantly between primary and secondary infection which were myalgia and gastrointestinal symptoms.

The most prevalent serotype was DENV-4 during 2001 and 2002, followed by DENV-1 and DENV-2 in 2003. Although seasonal variations in virologically confirmed dengue cases were observed, these should be interpreted with caution, because only a proportion of AUF patients were tested by RT-PCR. A previous surveillance study showed that all four DENV serotypes co-circulated in southern Vietnam in 2001 with isolation of DENV-2 and DENV-3 in Binh Thuan province.^20^ This study shows that all DENV serotypes have been circulating in Binh Thuan province, probably with a shift of the dominant DENV serotype during the study period. Co-circulation of all DENV serotypes is well described in Asia and indicates hyperendemicity.^21^

In discussing viraemia in relation to serotypes, it is important to point out that a primary immune response was biased towards DENV-1, which accounted for 87% of primary DENV infections. DENV-1 infections were characterized by significantly higher viraemia levels than DENV-4 infections and slightly higher than DENV-2 (Fig. 2D). DENV-1 infected patients are more likely to be younger than DENV-2 or DENV-4 infected patients. Age is an important risk factor for the development of severe dengue disease.^22^

The relationship between viraemia and host antibody response is less clear and has been the topic of many studies. In DENV-1 infections, viraemia was higher than in secondary infections but this pattern was not found for DENV-2 infections for which lower viraemia was associated with higher anti-DENV antibody titres.^18,23–25^ Interestingly, primary immune status was found in half of the symptomatic DENV-1 cases whereas 100% DENV-4 and almost all DENV-2 infections exhibited a secondary response. Likewise, primary infections with DENV-1 were predominant compared to other serotypes in a retrospective study of Thai dengue cases.^21^ Analysis of a co-epidemic with DENV-2 and DENV-4 indicated that the vast majority of DENV-2 infections were associated with a secondary immune response.^26^

DENV-2 viruses have most commonly been associated with DHF/DSS,^21,27,28^ along with DENV-1 and DENV-3 viruses.^29,30^ DENV-2 and DENV-4 have been associated with increased disease severity as a secondary infection, whereas DENV-1 and DENV-3 seem to cause more severe disease in primary infection than do the other two serotypes.^27,31^

These data should be interpreted against their own history of DENV co-circulation and herd immunity, which may be different between regions. In our study, clinical manifestations did not differ significantly among patients infected with different serotypes but this was a study population selected with only mild disease in a highly endemic area with a history of circulation of all DENV serotypes.

Our data showed sex differences with a male predominance. Reported sex differences are contradicting and differences in favour of males have been documented.^32,33^ The underlying causes of sex differences are not clear and multiple factors may play a role. A plausible explanation could be that there is a slight predominance in male births in Vietnam.^34^ A biased parents' health seeking behaviours towards males, differences in susceptibility and clinical presentation are other plausible causes.^35^

The strength of this study is the prospective enrolment of AUF patients over a period of five years which provides a comprehensive overview of epidemiological pattern over time at PHC in Vietnam. Only AUF patients were tested for dengue and, therefore, the study population included mildly symptomatic patients. Interestingly, a considerable amount (20%) of AUF patients were seen with primary DENV infection at PHC. Health seeking behaviour and the nature of study site (at PHC) may have caused the identification of more symptomatic primary dengue infections. This study was conducted shortly after a period of time in which malaria was the main cause of fever. Public awareness that malaria causes fever was high. As results of which, febrile patients presented themselves very early at PHC. We previously showed that patient delay was shorter for children, suggesting that parents are very concerned about the health of their children and take the opportunity to seek help as soon as possible.^36^ These patients are probably a true reflection of burden of symptomatic dengue in the general population.

A limitation of this study is that serotypes identification by RT-PCR showed a low yield. There are several possible explanations for this low detection rate. First, serum samples were aliquoted and stored at −20 °C up to one month. Samples were collected for transportation to CRH hospital for storage at −70 °C once monthly. DENV RNA could have degraded due to sub-optimal storage and transport conditions. Secondly, samples were used previously for other studies.^9,10,14,15,19^ It is possible that several freeze–thawing steps contributed to the degradation of DENV RNA. Thirdly, the majority of our study population presented very early in course of dengue and clinical manifestations were very mild. At this early stage of disease, viraemia levels may have been low.^31^

In conclusion, our data confirm earlier findings that dengue is highly endemic in southern Vietnam and shows that all four serotypes are prevalent.

## Figures and Tables

**Table 1:** Demographic data of patients with acute undifferentiated fever and dengue.

	Patients with AUF (*N* = 14512)	Serologically confirmed/total tested (*N* = 351/1938)	Virologically confirmed/total tested (*N* = 86/351)
Male/female (ratio)	1.28^a^ (8139/6373)	1.76 (224/127)	2.58 (62/24)
Median age (yr) (range)	18.3 (0.1–95.1)^b^	18.9 (4.2–73.7)	16.3(5.6–55.2)
Child/adult (ratio)	0.70	0.61	0.87

a Sex was unknown in 7 AUF patients.

b Age was unknown in 8 AUF patients.

**Table 2:** Frequency of symptoms and physical findings on admission in patients diagnosed with primary, secondary and children and adults.

Variables	Primary infection (*N* = 67^a^)	Secondary infection (*N* = 283)	*P*	Children 5–15 years (*N* = 132^a^)	Adult ≥15 years (*N* = 218)	*P*
Age (yr)^b^	12.2 (5.3–51.7)	20.6 (5.5–73.6)	<0.001	11.2 (5.3–15.0)	28.1 (15.5–73.7)	
Male/female	44/23	180/103		80/52	144/74	
Body temperature (^°^C)^b^	38.9 (38.0–39.9)	39.0 (38.0–42.0)		39.0 (38.0–42.0)	39.0 (38.0–40.0)	
Pulse pressure (mmHg)^b^	30.0 (30.0–60.0)	40.0 (20.0–60.0)	<0.001	30.0 (20.0–55.0)	40.0 (20.0–60.0)	<0.001
Heart rate (beats/min)^b^	90.0 (70.0–130.0)	90.0 (66.0–130.0)		95.0 (76.0–130.0)	90.0 (66.0–120.0)	<0.001

Symptoms, (%)						
Headache	91.0	94.7		92.4	95.0	
Myalgia	29.9	52.7	0.001	25.8	61.9	<0.001
Sore throat	32.8	42.4		44.7	38.1	
Backache	20.9	19.1		6.1	27.5	<0.001
Nausea	20.9	23.7		14.4	28.4	0.003
Cough	28.4	33.6		31.8	33.0	
Arthralgia	16.4	17.7		10.6	21.6	0.009
Gastrointestinal symptoms	25.4	13.1	0.022	12.1	17.4	

Findings at physical examination, (%)						
Pallor	19.4	17.3		12.1	21.1	0.043
Dehydration	11.9	13.1		13.6	12.4	
Pharyngitis	40.3	49.5		48.5	47.2	
Lymph nodes	0.0	0.7		1.5	0.0	
Rash	0.0	2.5		0.0	3.2	
Bruises	6.0	4.2		0.8	6.9	0.007

a One child (age = 4.2 years) was excluded from analysis.

b Median (range); differences between groups were analyzed with Mann–Whitney tests. Fisher's exact test was used in all dichotomous data. Gastrointestinal symptoms: abdominal pain, diarrhea and constipation.
